# Editorial: Management of osteoporosis in patients with chronic kidney disease

**DOI:** 10.3389/fmed.2022.1032219

**Published:** 2023-01-04

**Authors:** Mohamed Abdalbary, Mahmoud Sobh, Eman Nagy, Sherouk Elnagar, Nehal Elshabrawy, Rasha Shemies, Mostafa Abdelsalam, Kamyar Asadipooya, Alaa Sabry, Amr El-Husseini

**Affiliations:** ^1^Mansoura Nephrology and Dialysis Unit, Mansoura University, Mansoura, Egypt; ^2^Division of Endocrinology, University of Kentucky, Lexington, KY, United States; ^3^Division of Nephrology and Bone and Mineral Metabolism, University of Kentucky, Lexington, KY, United States

**Keywords:** CKD-MBD, transplantation, osteoporosis, trabecular bone score, vitamin D, FGF-23, machine learning, osteoprotegerin

Chronic kidney disease (CKD) is a major health problem that has devastating metabolic and bone consequences. Osteoporosis is one of the pivotal metabolic disorders in patients with CKD which can increase the risk of fractures and mortality. Most nephrologists are familiar with management of CKD-Mineral and Bone Disorders (CKD-MBD), however, there is a big gap in diagnosis and management of osteoporosis. This special issue editorial is trying to focus on identifying the mechanisms behind bone loss that will help to precisely improve the outcome of patients with CKD.

In terms of diagnostic tools, trabecular bone score (TBS) is an emerging analytical tool depends on the gray-level variations of lumbar vertebrae and can be applied to DEXA images to assess bone micro-architecture. Clinical importance of TBS has been proved in patients with osteoporosis. Though, its value in patients with end-stage kidney disease (ESKD) needs to be validated. Patients on maintenance dialysis had an altered bone microarchitecture, however, there are no prospective trials to evaluate TBS role in fracture prediction in ESKD. In this special issue, Poiana et al. reviewed the role of TBS in fracture risk assessment and management of CKD-MBD in dialysis patients. They concluded that TBS might add more information to DEXA measurements and improve the fracture risk assessment.

Cardiovascular disease is one of the catastrophic complications in patients with CKD. Both traditional and non-traditional risk factors contribute in the development of cardiovascular calcification (VC) (1). Osteoprotegerin (OPG) impedes bone loss through its inhibitory effect on osteoclast function. Its role as VC inhibitor is evolving (2), however, several studies reported a positive correlation between serum OPG and adverse cardiovascular outcomes (3–5). Possible explanation of this discrepancy is that the rise of OPG is a compensatory mechanism against factors that promote VC, atherosclerosis, and other forms of vascular damage (6). In a cross-sectional study by Okasha et al., the severity of VC increased in patients with advanced CKD. Additionally, they found that serum OPG and phosphorus levels were significant independent predictors of VC.

Vitamin D is crucial for regulation of bone and mineral metabolism (7). Calcidiol [25(OH)D] deficiency is a common finding in patients with CKD (8, 9). Treatment of calcidiol deficiency is a debatable topic and there is no strong evidence regarding the type and the dose of vitamin D as well as the targeted threshold for treatment (10). Alfacalcidol is a vitamin D receptor analog which is commonly used in patients on maintenance dialysis. Its inhibitory effect on the parathyroid hormone as well as bone turnover is well proved (11, 12). Vitamin D activation is not limited to the kidney and calcitriol is produced in extrarenal tissues as well (12). In a prospective randomized trial by Matuszkiewicz-Rowińska et al., 13 weeks of oral cholecalciferol (15,000 IU/week) was more effective than alfacalcidol (1.5 μg/week) in increasing both 25-(OH)D and 1,25(OH)D levels in patients on maintenance hemodialysis. Moreover, there were no significant differences in serum calcium, phosphate, iPTH, FGF-23, and sclerostin levels over the study period.

Postulating a hypothesis and testing it in suitable model is a fundamental step in understanding complex challenging medical problems as CKD-MBD. Traditionally, murine models were used for this purpose (13), however, extrapolating evidence from mouse to human pathophysiology has demonstrated multiple pitfalls. Mice show considerable genetic diversities in bone diseases. Additionally, large number of animals are needed to test multiple interventions. As an alternative, in this issue Gaweda et al., discussed the use of a human comprehensive mathematical tool known as quantitative systems pharmacology modeling. Human biochemical processes can be simulated explaining the interaction between multiple organs and biomarkers. Gaweda et al., validated their model using human data from the Chronic Renal Insufficiency Cohort (CRIC) study (14, 15). With continuous upgrading of this mathematical model, artificial intelligence would be a novel way of processing complicated medical data and replicating medical expertise. In the current issue the authors explore the most recent advances in using artificial intelligence in CKD management.

FGF-23, a phosphaturic hormone secreted by osteocytes, and its co-receptor klotho have gained much interest in patients with CKD (16). FGF-23-α-Klotho pathway links CKD-MBD, kidney function, and cardiovascular disease. With loss of kidney function, FGF-23 levels increase, while α-Klotho levels decrease (17). While increase FGF-23 levels are associated with left ventricular hypertrophy, atherosclerosis, and inflammation (17, 18), α-Klotho has an anti-apoptotic, anti-senescence and anti-fibrotic effects (19). Less is known about FGF-23-α-klotho pathway in kidney transplant recipients (KTRs) and kidney donors. In KTRs, as in patients with CKD, cardiovascular disease is the main cause of death. Moreover, MBD derangements continue after kidney transplantation. On the other side, living kidney donors did not have an increase in cardiovascular diseases but they may have increased risk of ESKD (20). α-Klotho levels remain lower than baseline at least 1 year after kidney donation (21). Long term metabolic sequences in kidney donors are not clearly defined. Furthermore, the potential therapeutic intervention of FGF-23-α-Klotho pathway is an interesting field which needs to be explored. In this issue, Gupta et al. are summarizing the up-to-date knowledge of FGF-23 and α-Klotho in KTRs and living kidney donors and highlighting the prospective role of this pathway in patients' management in the future.

Bone and mineral disorders are common in KTRs with increased risk of fractures. Moreover, management of CKD-MBD in KTRs is challenging due to lack of randomized clinical trials and national/international guidelines. CKD-MBD in KTRs are related to several factors including steroid usage, persistent hyperparathyroidism, low 25-OH-vitamin D as well as high FGF23 which may result in low phosphorus with defective bone formation and mineralization. Some studies demonstrated that hyperparathyroidism is the most predominant renal osteodystrophy (ROD) form (22, 23), however, several recent studies revealed that normal and low bone turnover are the commonest form of ROD in KTRs (24–28). Molinari et al., reviewed the possible pathogenesis, biochemical abnormalities, and impact of post-transplant MBD. Additionally, they designed an informative algorithm for post-transplant MBD management.

## Author contributions

All authors listed have made a substantial, direct, and intellectual contribution to the work and approved it for publication.
